# The Impact of Bariatric Surgery on Weight Reduction and the Resolution of Comorbidities in Older Geriatric Populations of Saudi Arabia: A Retrospective Study

**DOI:** 10.7759/cureus.69349

**Published:** 2024-09-13

**Authors:** Abdulrahman M Alamri, Saeed A Alsareii, Nadia A Isaway, Saleh H Alshaiban, Saleh Y Alyami, Mustafa T Alsaid

**Affiliations:** 1 Department of Surgery, College of Medicine, Najran University, Najran, SAU; 2 Department of Medicine, College of Medicine, Najran University, Najran, SAU; 3 Department of Surgery, Najran Military Hospital, Najran, SAU

**Keywords:** retrospective study, geriatrics, comorbidities, weight reduction, bariatric surgery

## Abstract

Background

Obesity is a significant health concern among older adults, leading to various comorbidities and reduced quality of life. Bariatric surgery (BS) has emerged as a potential intervention, but its efficacy in geriatric populations, particularly in Saudi Arabia, is not well-established.

Aims

This retrospective study aims to evaluate the impact of BS on weight reduction and comorbidity resolution in Saudi Arabian geriatric populations.

Methods

A retrospective cohort study was conducted at King Khalid Hospital, Saudi Arabia, involving geriatric patients aged 60 and above who underwent BS between January 2018 and December 2022. Data were collected from medical records and analyzed using descriptive statistics, chi-square tests, t-tests, and multivariate regression analysis.

Results

The study included a total of 26 patients with a mean age of 64 years. Of these, 18 (69.3%) were females, while eight (30.7%) were males, and 23 (87%) underwent sleeve gastrectomy (SG), while three (13%) had Roux-en-Y gastric bypass (RYGB). Preoperative comorbidities majorly included diabetes (17, 35.42%), hypertension (11, 22.92%), and anemia (four, 8.33%). The average body mass index (BMI) of the patients decreased significantly from 45.12 to 37.29 at three months and further to 31.36 at six months post surgery. Total weight loss (TWL) was 19.92% at three months and 35.15% at six months, while the percentage of excess weight loss (%EWL) was 33.42% at three months and 57.85% at six months. Results also showed a significant reduction in the number of comorbidities postoperatively. A significant association with gender, preoperative weight, and preoperative height at three and six months and a significant association with preoperative BMI and comorbidity status at six months were recorded.

Conclusion

The study suggests that bariatric surgery is effective in achieving significant weight loss and improving comorbidities in geriatric patients. Few demographic and clinical features affect the outcome of the weight loss.

## Introduction

Obesity is a multifaceted condition characterized by an excessive accumulation of body fat, leading to adverse effects on physical health and placing significant financial burdens on healthcare systems [1]. The impact of obesity is especially severe among older individuals, who often face additional health challenges such as type 2 diabetes, hypertension, cardiovascular disease, HIV infection, and musculoskeletal disorders [2-4]. These coexisting conditions, known as comorbidities, not only reduce the quality of life but also increase the risk of premature mortality [4].

The rise in obesity rates globally, especially among older individuals, is a concerning trend [5]. In Brazil, a long-term study found that overweight was the most common nutritional status among older adults, affecting between 33.75% and 41.38% of the population over 15 years [6]. In China, approximately 120 million people aged 45 and older suffer from obesity-related hypertension, with a prevalence of 22.7% [7]. In India, the prevalence of overweight and obesity is higher in older females, with rates of 23.2% and 15.5%, respectively, compared to males [8]. These global findings emphasize the urgent need for targeted interventions to address the rising rates of obesity among older populations worldwide. In Saudi Arabia, obesity among the geriatric population is a significant health concern, as highlighted by various studies [9,10]. A comprehensive geriatric assessment conducted in primary healthcare centers across the country revealed a high prevalence of obesity, with 22.2% of older people affected [9]. Another study that also focused on the adult population reported an obesity prevalence of 23%, with variations by gender [10]. These findings align with global trends of increased prevalence of obesity among older adults, emphasizing the need for effective interventions.

Bariatric surgery (BS), which encompasses a spectrum of procedures aimed at reducing stomach size or altering digestive pathways, has emerged as a viable intervention, especially for those who have not achieved significant weight loss through conventional modalities such as dietary adjustments and physical activity facilitating weight loss and often resolving comorbidities [2,11]. While the benefits of BS in general populations are well-documented in several studies [2,11,12], research on its efficacy in geriatric populations, particularly within the Saudi Arabian context, remains scarce. Hence, this retrospective analysis endeavors to bridge this knowledge gap by examining the effects of BS on weight reduction and comorbidity resolution in elderly populations aged 60 and above who underwent BS in Saudi Arabia.

## Materials and methods

Study design and settings

A retrospective cohort study was conducted to investigate the outcomes of bariatric surgery in Saudi Arabian geriatric patients [13]. The study utilized existing medical records and databases from King Khalid Hospital in the southwestern region of Saudi Arabia.

Study population

The study population consisted of Saudi Arabian geriatric patients aged 60 years and above who underwent bariatric surgery between January 2018 and December 2022 at King Khalid Hospital. Patients' records were included if they met the following criteria: aged 60 years or older, underwent any type of bariatric surgery, and had complete medical records, including three-month and six-month follow-up records. Conversely, patients were excluded if they had missing or incomplete medical records, a history of previous bariatric surgery, or undergone bariatric surgery outside of the specified time frame and study area.

Data collection and analysis

Data were collected from the electronic medical records of King Khalid Hospital in the southwestern region of Saudi Arabia after appropriate permissions and ethical considerations were sorted. Data collection included patient demographics (age and gender), preoperative comorbidities (including anemia, diabetes, asthma, hypertension, knee pain, hypothyroidism, depression, obstructive sleep apnea, arthritis, sleep apnea with restrictive airway disease, heart disease, and osteoarthritis), postoperative comorbidities (including diabetes, heartburn, hypothyroidism, hypertension, and vomiting), body mass index (BMI) and weight measurements, type of bariatric surgery performed (Roux-en-Y gastric bypass {RYGB} and sleeve gastrectomy {SG}), and follow-up data at three and six months postoperatively. Patients with incomplete data sets were excluded from the study, and all patient data, including demographic information, weight, BMI, and comorbidities, were extracted from electronic medical records and stored in a secure, password-protected database. Descriptive statistics (mean and standard deviation, frequency, and percentage) were used to summarize the demographic and clinical characteristics of the study population. Inferential statistics were employed to examine the relationships between variables. Specifically, t-tests were used to compare continuous variables, such as BMI and weight measurements, pre- and postoperatively, and analysis of variance (ANOVA) was used to compare means of continuous variables across more than two groups. All statistical analyses were performed using SPSS (IBM SPSS Statistics, Armonk, N). A p-value of less than 0.05 was considered statistically significant. A Shapiro-Wilk test was done on post-BMI three months and post-BMI six months with p-values of 0.53 and 0.24, showing that data were normally distributed.

Ethical clearance

Ethical clearance was obtained prior to the commencement of data collection from the Research Ethics Committee of Najran University, Saudi Arabia, with an institutional review board (IRB) approval number of 202405-076-020754-047551. Patient confidentiality was ensured throughout the study, and all data were anonymized and stored securely.

## Results

Demographic and clinical characteristics

The study identified 26 geriatric patients who underwent bariatric surgery, with a mean age of 64 years. Among them, 18 (69.3%) were female, and eight (30.7%) were male. The majority (23, 87%) underwent sleeve gastrectomy (SG), while three (13%) underwent Roux-en-Y gastric bypass (RYGB). Preoperatively, the patients had a total of 48 comorbidities, with diabetes being the most common (17, 35.42%), followed by hypertension (11, 22.92%) and anemia (three, 8.33%). Postoperatively, the total number of comorbidities decreased to 16, with significant reductions in diabetes and hypertension cases (Table 1).

**Table 1:** Demographic and Clinical Characteristics of Geriatric Patients Undergoing Bariatric Surgery The table was presented as categories, subcategories, frequencies (number of cases), and percentage RYGB, Roux-en-Y gastric bypass; SG, sleeve gastrectomy

Category	Subcategory	Frequency	Percentage
Age	Above 64	11	42.3
	Below 64	15	57.7
	Mean Age: 64		
Gender	Male	8	30.7
	Female	18	69.3
Surgery	RYGB	3	13.0
	SG	23	87.0
Comorbidity (Preoperative)	Anemia	4	8.33
	Diabetes	17	35.42
	Asthma	3	6.25
	Hypertension	11	22.92
	Knee Pain	1	2.08
	Hypothyroidism	4	8.33
	Depression	1	2.08
	Obstructive Sleep Apnea	3	6.25
	Arthritis	1	2.08
	Sleep Apnea With Restrictive Airway Disease	1	2.08
	Heart Disease	1	2.08
	Osteoarthritis	1	2.08
Comorbidity (Postoperative)	Diabetes	7	43.7
	Heartburn	1	6.25
	Hypothyroidism	2	13.5
	Hypertension	5	31.2
	Vomiting	1	6.25

Changes in BMI, total weight loss (TWL), and percentage of excess weight loss (%EWL)

The average BMI of patients decreased significantly from 45.12 to 37.29 at three months and further to 31.36 at six months post surgery. Specifically, RYGB patients saw a BMI reduction from 46.23 to 38.31 at three months and to 29.84 at six months. SG patients showed a decrease from 44.97 to 37.16 at three months and to 31.58 at six months. Total weight loss (TWL) was 19.92% at three months, increasing to 35.15% at six months. For RYGB patients, TWL increased from 19.66% at three months to 41.00% at six months. SG patients saw an increase from 19.96% at three months to 34.39% at six months. The percentage of excess weight loss (%EWL) increased from 33.42% at three months to 57.85% at six months. RYGB patients had an increase from 31.81% to 67.63%, and SG patients saw an increase from 33.63% to 56.58% over the same period (Table 2).

**Table 2:** Changes in BMI, Total Weight Loss (TWL), and Percentage of Excess Weight Loss (%EWL) Following Post-operation ^a^Compared to baseline ^b^Six months compared to three months *Significant level at p < 0.05 **Significant level at p < 0.01 BS, bariatric surgery; RYGB, Roux-en-Y gastric bypass; SG, sleeve gastrectomy; BMI, body mass index

	BMI			TWL		%EWL	
	Baseline	3 Months	6 Months	3 Months	6 Months	3 Months	6 Months
BS	45.12 ± 1.45	37.29 ± 1.16^a^	31.36 ± 0.91^ab^	19.92 ± 1.83**	35.15 ± 3.25**	33.42**	57.85**
RYGB	46.23 ± 5.23	38.31 ± 3.58	29.84 ± 2.4^ab^	19.66 ± 5.54	41.00 ± 7.21*	31.81*	67.63*
SG	44.97 ± 1.55	37.16 ± 1.26^a^	31.58 ± 0.99^ab^	19.96 ± 1.99**	34.39 ± 3.57**	33.63**	56.58**

Resolution of comorbid conditions

The average number of comorbid conditions per patient decreased significantly from 1.84 preoperatively to 0.65 postoperatively. RYGB patients saw a reduction from 1.66 to 0.66 and SG patients from 1.86 to 0.65 (Table 3).

**Table 3:** Resolution of Comorbid Conditions, Preoperative Versus Postoperative ^a^Statistical significance at p < 0.05 when compared to preoperative values BS, bariatric surgery; RYGB, Roux-en-Y gastric bypass; SG, sleeve gastrectomy

Comorbidity		
	Preoperative	Postoperative
BS	1.84 ± 0.21	0.65 ± 0.14^a^
RYGB	1.66 ± 0.88	0.66 + 0.66
SG	1.86 ± 0.22	0.65 ± 0.14^a^

Impact of demographic and clinical features on total weight loss

At three months, significant associations were found between total weight loss and gender, preoperative weight, and height. Males, heavier patients, and taller patients showed higher weight loss (Table 4). At six months, significant associations were found with gender, preoperative BMI, weight, height, and comorbidity status. Male patients, as well as patients with higher preoperative BMI, weight, and height, showed greater weight loss (Table 5).

**Table 4:** Impact of Demographic and Clinical Features on Total Weight Loss at Three Months Significant level at p < 0.05 SD, standard deviation; RYGB, Roux-en-Y gastric bypass; SG, sleeve gastrectomy; BMI, body mass index

Category	Subcategory	Mean	SD	t/F value	P-value
Gender	Female	17.11	8.08	0.5	0.01
	Male	26.25	9.42		
Age	Below 64	21.44	10.63	3.04	0.34
	Above 64	17.86	7.28		
Preoperative (Number of Comorbidities)	Above 2	19.81	8.11	2.77	0.94
	Below 2	20.10	11.57		
Preoperative (Comorbidity Status)	Presence	19.30	9.07	0.36	0.36
	Absence	24.6	12.34		
Types of Bariatric Surgery	RYGB	19.66	9.60	0.06	0.96
	SG	19.96	9.55		
Preoperative BMI	Above 45.12	24.00	8.80	0.001	0.05
	Below 45.12	16.94	8.87		
Preoperative Weight	Above 111.9	25.00	7.88	0.34	0.001
	Below 111.9	14.00	7.41		
Preoperative Height	Above 157.5	26.41	7.97	0.16	0.001
	Below 157.5	15.16	7.34		

**Table 5:** Impact of Demographic and Clinical Features on Total Weight Loss at Six Months Significant level at p < 0.05 SD, standard deviation; RYGB, Roux-en-Y gastric bypass; SG, sleeve gastrectomy; BMI, body mass index

Category	Subcategory	Mean	SD	t/F value	P-value
Gender	Female	30.67	13.42	0.10	0.03
	Male	45.25	19.50		
Age	Below 64	34.31	13.24	0.97	0.83
	Above 64	35.77	19.15		
Preoperative (Number of Comorbidities)	Above 2	36.38	13.47	2.06	0.64
	Below 2	33.2	21.39		
Preoperative (Comorbidity Status)	Presence	32.7	13.97	2.63	0.03
	Absence	54.00	26.51		
Types of Bariatric Surgery	RYGB	41.00	12.49	0.40	0.52
	SG	34.39	17.15		
Preoperative BMI	Above 45.12	45.18	14.61	0.68	0.006
	Below 45.12	27.80	14.27		
Preoperative Weight	Above 111.9	46.21	12.53	0.003	0.000
	Below 111.9	22.25	10.24		
Preoperative Height	Above 157.5	43.96	16.82	0.04	0.01
	Below 157.5	26.70	13.61		

## Discussion

These study results offer a comprehensive understanding of the impact of BS on weight reduction and comorbidity resolution in the Saudi Arabian geriatric population attending King Khalid Hospital, Saudi Arabia. It sheds light on the demographic and clinical characteristics of geriatric patients, as well as the changes in BMI, weight loss, and comorbidities post surgery. Overall, the study's results demonstrate the efficacy of bariatric surgery in geriatric patients, with significant improvements in BMI, weight loss, and comorbidity resolution. The findings also highlight the importance of considering demographic and clinical factors when predicting weight loss outcomes in this population.

Our study involving 26 geriatric patients with a mean age of 64 years demonstrates that bariatric surgery is both safe and effective for older adults. These findings counter concerns about the risks and efficacy of bariatric surgery in older patients who often have more comorbidities and reduced physiological reserves [14,15], aligning with existing literature supporting its benefits in this age group [16,17]. Our study's results add to the growing body of evidence supporting the safety and efficacy of BS in geriatric patients. By debunking the myth that BS is contraindicated in older adults, we can provide more patients with access to this life-changing treatment.

The majority of patients in this study were female (18, 69.3%), which is consistent with previous studies showing a higher prevalence of bariatric surgery among females, possibly due to a greater willingness to seek surgical weight loss interventions among females compared to males [18,19]. Furthermore, results showed a high percentage of patients undergoing SG (23, 87%), reflecting a trend in preference for SG observed in other studies due to its relatively lower complication rates, technical simplicity, and effectiveness in long-term weight reduction [20,21].

The results of this study demonstrate the efficacy of bariatric surgery in achieving significant weight loss among geriatric patients, aligning with the consensus of existing literature. Our findings corroborate previous studies that have consistently shown that bariatric surgery is a safe and effective treatment option for older adults, leading to substantial weight loss and improvement in obesity-related comorbidities [16,20,22-24]. The consistency of these findings across various studies reinforces the notion that bariatric surgery can be a valuable treatment option for geriatric patients struggling with obesity and highlights the importance of considering age as a factor in treatment decisions.

The significant reduction in BMI from 45.12 preoperatively to 31.36 at six months postoperatively as seen in this current study underscores the surgery's role in managing obesity in older adults. This decrease in BMI is particularly noteworthy given the challenges associated with weight gain in the geriatric population, which often include poor energy balance and a higher prevalence of comorbidities [1,25]. Similarly, the increase in total weight loss from 19.92% at three months to 35.15% at six months indicates that the benefits of bariatric surgery extend beyond the immediate postoperative period. This sustained weight loss is critical for older adults, as it can lead to improvements in physical function, mobility, and overall quality of life [26]. Weight reduction in this population is associated with a decreased risk of cardiovascular diseases, improved glucose metabolism, and better management of conditions such as hypertension and osteoarthritis [23,26].

Moreover, the percentage of excess weight loss, which increased from 33.42% at three months to 57.85% at six months, demonstrates that patients not only lost weight but also achieved a significant reduction in their excess weight. This metric is essential in evaluating the success of bariatric surgery, as it reflects the extent to which patients are approaching a healthier weight range. Excess weight loss is a key predictor of improved health outcomes, including reduced mortality rates and lower incidence of obesity-related diseases [27,28]. The positive outcomes observed in this study suggest that bariatric surgery should be considered a viable option for weight management in geriatric patients. However, other postoperative care including nutritional counseling, physical therapy, and regular follow-ups to ensure sustained weight loss and overall health improvement is essential.

This current study compared the effectiveness of two bariatric surgeries, Roux-en-Y gastric bypass (RYGB) and sleeve gastrectomy (SG), in reducing BMI and promoting weight loss. The results showed that both surgeries led to significant BMI reduction and weight loss, indicating that both procedures are viable options for patients seeking weight loss solutions. However, there were notable differences between the two surgical procedures. RYGB patients experienced a more pronounced BMI reduction and greater weight loss compared to SG patients over six months. Specifically, RYGB patients had a higher total weight loss (TWL) and percentage of excess weight loss (%EWL) compared to SG patients. Furthermore, the study found that RYGB patients showed a more substantial weight loss trajectory, with a higher initial TWL at three months and a greater increase by six months. This suggests that RYGB may offer a more effective solution for patients seeking greater weight loss outcomes. These outcomes are in line with the existing literature, which highlights that both RYGB and SG are effective in promoting significant weight loss, though RYGB often results in slightly greater weight reduction [29,30].

In continuation, findings from our study reveal a significant reduction in comorbid conditions following bariatric surgery, with a decrease in the average number of comorbidities per patient from 1.84 preoperatively to 0.65 postoperatively. This dramatic decrease in comorbid conditions indicates that bariatric surgery can lead to substantial improvements in obesity-related comorbidities, particularly diabetes and hypertension as corroborated by numerous studies [16,20,22-24]. Preoperatively, our patients presented with 48 comorbidities, with diabetes (17, 35.42%), hypertension (11, 22.92%), and anemia (four, 8.33%) being the most common. The high prevalence of these conditions is consistent with the literature, which indicates that obesity is frequently associated with multiple comorbidities, including metabolic disorders such as diabetes and hypertension [1,31]. However, postoperative findings revealed that the total number of comorbidities decreased to 16, showcasing significant improvements, particularly in diabetes and hypertension cases. This reduction in comorbidities is well-documented in the literature, where studies have shown that bariatric surgery can lead to significant remission of type 2 diabetes and reduction in cardiovascular risk factors [32,33].

Our study also analyzed the impact of demographic and clinical features on total weight loss at three and six months post surgery. At three months, significant associations were found between total weight loss and gender, preoperative weight, and height, with males, heavier patients, and taller patients showing higher weight loss. At six months, additional significant associations included preoperative BMI, weight, height, and comorbidity status, indicating that males; patients with higher preoperative BMI, weight, and height; and those without comorbidities experienced greater weight loss.

These findings align with existing literature, which suggests that demographic and clinical factors can influence weight loss outcomes post-bariatric surgery. A study revealed that males often experience greater weight loss compared to females, possibly due to differences in muscle mass and metabolic rates [34]. However, contradictory findings were seen with studies that showed females had better effects [35] and those that did not record any significant difference [36]. Additionally, higher preoperative BMI and weight have been associated with more significant weight loss post surgery, as patients with more weight to lose tend to see more pronounced reductions [37]. The absence of comorbidities may also contribute to better weight loss outcomes, as healthier patients are likely to have better postoperative recovery and adherence to lifestyle changes [38].

Study limitations

While this retrospective study provides valuable insights into the impact of bariatric surgery on weight reduction and comorbidity resolution in Saudi Arabian geriatric populations, several limitations must be acknowledged. The study involved a relatively small sample size of 26 patients, which may limit the generalizability of the findings. A larger sample would provide more robust data and enhance the reliability of the results. Future studies should aim to include a broader patient cohort to increase statistical power.

The study only included follow-up data at three and six months post surgery. While these time frames offer a snapshot of the early outcomes of bariatric surgery, they may not capture the long-term sustainability of weight loss and the resolution of comorbidities. Long-term follow-up is necessary to assess the durability of the benefits observed and to monitor potential late complications or weight regain. This study was conducted at a single medical center (King Khalid Hospital), which may limit the external validity of the findings. The outcomes observed may be influenced by the specific practices, expertise, and patient population of this hospital. Multicenter studies would help to ensure that the results are more broadly applicable to different clinical settings and populations.

## Conclusions

This study provides evidence supporting the efficacy of bariatric surgery in geriatric patients, highlighting significant reductions in BMI, total weight loss, and percentage of excess weight loss, alongside substantial improvements in comorbid conditions. Both RYGB and SG are effective surgical options, with RYGB showing slightly superior outcomes in weight loss metrics. The reduction in comorbid conditions post surgery is particularly noteworthy, reflecting the broader health benefits of bariatric procedures. Furthermore, demographic and clinical characteristics such as gender, preoperative BMI, weight, height, and comorbidity status significantly influence weight loss outcomes, underscoring the importance of personalized surgical interventions and postoperative care. These findings contribute to the growing body of literature affirming the role of bariatric surgery in improving health and the quality of life for elderly patients with obesity and related comorbidities. It is, however, important to note that the short follow-up period and the small sample size are limitations of this study, affecting its robustness and generalization.
